# High Intrinsic Aerobic Capacity Is Associated With a Distinct Epigenetic and Signaling Profile in the Aged Rat Brain

**DOI:** 10.1111/acel.70622

**Published:** 2026-07-14

**Authors:** Lei Zhou, Soroosh Mozaffaritabar, Erika Koltai, Takuji Kawamura, Mitsuru Higuchi, Sylwester Kujach, Yaodong Gu, Lauren Gerard Koch, Steven Loyal Britton, Zsolt Radák

**Affiliations:** ^1^ Research Institute of Molecular Exercise Science Hungarian University of Sports Science Budapest Hungary; ^2^ Faculty of Sport Sciences Waseda University Tokorozawa Japan; ^3^ Department of Physiology Gdansk University of Physical Education and Sport Gdansk Poland; ^4^ Faculty of Sport Science Ningbo University Ningbo China; ^5^ Department of Physiology and Pharmacology The University of Toledo College of Medicine and Life Sciences Toledo Ohio USA; ^6^ Department of Anesthesiology University of Michigan Ann Arbor Michigan USA

**Keywords:** aging, brain, DNA methylation, inflammation, intrinsic aerobic capacity, RRBS, VO_2_max

## Abstract

Exercise is a powerful non‐pharmacological strategy for preserving brain health during aging. However, whether intrinsic exercise capacity is associated with a distinct molecular phenotype in the aged brain, independent of training intervention, remains unclear. Aged selectively bred low‐capacity runner (LCR) and high‐capacity runner (HCR) rats were studied. Hippocampal DNA methylation was profiled by reduced representation bisulfite sequencing (RRBS), and differentially methylated regions (DMRs) were annotated and functionally enriched. Spatial learning, aerobic capacity, and cortical protein signaling were assessed by Morris water maze, VO_2_max testing, and Western blotting. RRBS identified 6452 significant DMRs, most of which were hypermethylated in HCR rats (82.4%) and enriched in open‐sea and gene‐body regions. Genes linked to hypermethylated DMRs showed context‐dependent enrichment, particularly in intronic and exonic regions, highlighting MAPK, PI3K‐Akt, TNF, calcium, and synaptic signaling pathways. Core methylation‐related enzymes TET1/2 and DNMT3A/B were unchanged. Cortical protein profiling showed higher phosphorylation of ERK1/2, AKT, mTOR, S6, and synapsin in HCR rats, together with higher JNK2, p38, NF‐κB, TNF‐α, and OGG1 abundance, whereas protein carbonylation and selected exercise‐responsive neurotrophic and metabolic/mitochondrial markers were unchanged. Exploratory correlation analysis identified a subset of DMRs associated with individual VO_2_max values. Morris water maze performance did not differ significantly between groups. High intrinsic exercise capacity in aged rats is associated with distinct hippocampal DNA methylation patterns that align with cortical protein profiles involving MAPK, AKT‐mTOR‐S6, synaptic, and inflammation‐related signaling. These findings suggest a distinct molecular phenotype linked to intrinsic aerobic capacity in the aged brain.

## Graphical abstract

High intrinsic aerobic capacity in aged rats is associated with a hypermethylation‐enriched hippocampal DMR profile, mainly involving open‐sea and gene‐body‐associated regions, together with cortical MAPK, AKT‐mTOR‐S6, synaptic, and inflammation‐related protein signaling changes.

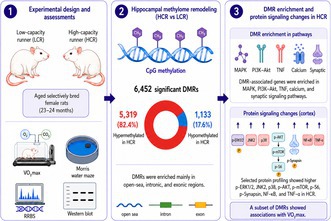

## 1 Introduction

Aging is accompanied by a progressive decline in brain function, characterized by impaired synaptic plasticity, altered neuronal signaling, increased inflammatory tone, and greater susceptibility to cognitive dysfunction and neurodegenerative processes (Sun et al. 2025; Wyss‐Coray 2016). Physical exercise is widely recognized as a beneficial non‐pharmacological strategy for maintaining brain health during aging (Hillman et al. 2008; Tari et al. 2025). A large body of work in humans and rodent models has shown that exercise training can modulate neuroplasticity, metabolic and vascular function, and inflammatory balance in the aging brain (Boa Sorte Silva et al. 2024; Tari et al. 2025). However, most previous work has focused on the effects of exercise interventions, addressing whether exercise exposure can modify brain aging. These studies do not directly answer whether aerobic capacity itself is associated with a distinct molecular state in the aged brain.

The selectively bred LCR and HCR rat model provides a valuable framework for addressing this question (Koch and Britton 2001). This model has been extensively used to investigate the biological consequences of intrinsic aerobic capacity, including its relationships with metabolic regulation, cardiovascular health, disease susceptibility, and lifespan (Koch et al. 2011; Wisløff et al. 2005). Cardiorespiratory fitness is a strong predictor of morbidity and mortality in humans and is increasingly viewed as an integrative marker of whole‐body health (Kodama et al. 2009). Consistent with this concept, intrinsic aerobic capacity has been linked to divergent aging trajectories across multiple systems (Koch et al. 2011; Wisløff et al. 2005). At the neural level, previous studies have suggested that HCR animals exhibit more favorable cognitive and neural characteristics than their LCR counterparts (Guzzoni et al. 2025; Kugler et al. 2024; Mäkinen et al. 2023). Nevertheless, the molecular features of such differences in the aged brain remain insufficiently defined.

Among candidate mechanisms, DNA methylation represents a stable but dynamic epigenetic layer through which inherited traits and long‐term physiological states may be integrated (Cavalli and Heard 2019). In the brain, DNA methylation contributes to neuronal identity, synaptic organization, and activity‐dependent transcriptional regulation (Halder et al. 2016; Lister et al. 2013). Yet it remains unclear whether intrinsic aerobic capacity in aged animals is associated with distinct brain methylation patterns, whether such patterns preferentially occur in promoter or non‐promoter genomic regions, and whether they are accompanied by corresponding differences in protein signaling pathways.

Therefore, the present study examined aged HCR and LCR rats to determine whether intrinsic exercise capacity is associated with differences in brain DNA methylation, signaling protein profiles, and behavioral phenotype. By doing so, this study aimed to distinguish inherited capacity‐associated brain features from those induced by exercise training and to clarify whether high intrinsic aerobic capacity is linked to a simple protective phenotype or to a more complex pattern of molecular remodeling in the aged brain.

## 2 Methods

### 2.1 Ethics Statement

All animal procedures were performed at the Research Institute of Molecular Exercise Science, Hungarian University of Sports Science (Budapest, Hungary). Experiments complied with the Guiding Principles for the Care and Use of Laboratory Animals, and relevant Hungarian regulations, and were approved by the National Animal Experimentation Scientific Ethics Council (Hungary) (approval No: PE/EA/62‐2/2021).

### 2.2 Experimental Animals

Selectively bred low‐ and high‐running capacity rats (LCR and HCR; 44th generation) were used. The animals were derived from genetically heterogeneous N:NIH stock and divergently selected for intrinsic running capacity. Female rats aged 23–24 months were included in the study (LCR, *n* = 6; HCR, *n* = 6). Animals were housed in temperature‐controlled rooms under a 12:12‐h light–dark cycle with ad libitum access to standard chow and water. All animals were maintained under standard cage conditions without access to running wheels or structured exercise training. All animals were used for physiological testing, behavioral assessment, and tissue collection. Genome‐wide RRBS analysis was initially performed on all animals; however, two HCR samples failed RRBS quality control criteria and were excluded from downstream methylation analyses, yielding a final RRBS cohort of HCR *n* = 4 and LCR *n* = 6.

### 2.3 Maximal Oxygen Uptake (VO_2_max) Testing

Cardiorespiratory fitness was assessed by measuring maximal oxygen uptake (VO_2_max) on a motor‐driven treadmill in a closed metabolic chamber customized for rats (Columbus Instruments, USA). VO_2_max values were normalized to body mass and expressed as mL/kg/min. Animals were acclimated to treadmill running for two consecutive days (5–10 min/day, 5–25 m/min). For VO_2_max testing, rats rested on the belt for 5 min and then started running at 5 m/min, with speed increased by 5 m/min every 2 min until exhaustion. VO_2_max was recorded when any of the following criteria were met: (i) a plateau in VO_2_ despite increasing speed, (ii) inability to maintain running posture, or (iii) respiratory quotient (VCO_2_/VO_2_) > 1 (Kawamura et al. 2025).

### 2.4 Morris Water Maze

Spatial learning performance was evaluated using the Morris water maze. Rats underwent acquisition training over four consecutive days, and escape latency was recorded for each training day. The maze apparatus, hidden platform location, water temperature, number of trials per day, and inter‐trial interval were as previously described (Bakonyi et al. 2023). Behavioral performance was compared between LCR and HCR rats to assess whether intrinsic exercise capacity was associated with altered learning ability during aging. Escape latency across training days was analyzed using repeated‐measures two‐way ANOVA with group and time as factors.

### 2.5 Tissue Collection and DNA Extraction

Rats were deeply anesthetized and tissues were rapidly excised. Cortex and hippocampus samples were snap‐frozen and stored at −80°C until further analysis. For RRBS analysis, total genomic DNA was extracted from hippocampal tissue using the PureLink Genomic DNA Mini Kit (Thermo Fisher Scientific, USA) according to the manufacturer's instructions. The hippocampus was prioritized for RRBS because of its relevance to spatial learning, aging‐related plasticity, and epigenetic regulation. Cortical tissue was used for protein profiling because sufficient hippocampal tissue was not available for parallel Western blot analyses and because cortical signaling provides a complementary pathway‐level readout of broader brain network‐level molecular remodeling.

### 2.6 RRBS Library Preparation and Sequencing

RRBS libraries were constructed using the Premium RRBS Kit V2 (Diagenode, Seraing, Belgium). Briefly, 100 ng of genomic DNA underwent MspI digestion, followed by end‐repair, adapter ligation, and size selection via Agencourt AMPure XP beads. Library concentrations were determined by qPCR, and eight samples were pooled in equimolar ratios using the manufacturer's Excel‐based pooling tool. The resulting pools were subjected to bisulfite conversion, purification, and PCR amplification according to the manufacturer's instructions. Final library quality was assessed using the Qubit dsDNA HS Assay (Life Technologies) for quantification and a Bioanalyzer High‐Sensitivity DNA chip for size distribution analysis. Sequencing was performed on an Illumina HiSeq 2500 system in paired‐end mode.

### 2.7 DNA Methylation Data Processing and Alignment

Raw Reduced Representation Bisulfite Sequencing (RRBS) reads were first subjected to quality control using FastQC and aggregated with MultiQC. To remove low‐quality bases and adapter sequences, Trim Galore (v0.6.10) was employed in RRBS‐specific mode (‐‐rrbs) and paired‐end mode (‐‐paired). Bases with a Phred quality score below 20 were trimmed. Cleaned reads were aligned to the *Rattus norvegicus* reference genome (mRatBN7.2/rn7) using Bismark with Bowtie2 as the underlying aligner. Methylation calls were extracted using the bismark_methylation_extractor with the ‐‐no_overlap parameter. The resulting coverage files were used for downstream statistical analysis. Because no separate library preparation or sequencing batch was present, no batch‐effect correction was applied.

### 2.8 Identification of Differentially Methylated Regions (DMRs)

All downstream methylation analyses were conducted in R using the methylKit package. CpGs with low coverage (< 5×) or extremely high coverage (> 99.9th percentiles) were filtered out to mitigate PCR bias. Following sequencing and PCA‐based quality control, two HCR samples failed RRBS quality criteria and were excluded from all subsequent methylation analyses, resulting in a final RRBS dataset of HCR *n* = 4 and LCR *n* = 6. Region‐level signals were quantified using a tiling‐window strategy with non‐overlapping 1 kb windows (window size = 1000 bp; step size = 1000 bp) (Liu et al. 2020). Tiles were retained if they contained at least 3 covered CpGs. Methylation calls were unified across samples using unite (minimum 3 samples per group required per tile). Differential methylation analysis between groups (HCR vs. LCR) was performed using the Chi‐square test implemented in calculateDiffMeth with an overdispersion correction (“MN”). Significant DMRs were identified based on FDR < 0.1 and a minimum methylation difference of 5%.

### 2.9 Genomic Annotation and Functional Enrichment

Significant DMRs were annotated to genomic features (promoters, exons, introns, 1–5 kb upstream, 5′UTR, 3′UTR) and CpG contexts (islands, shores, shelves, open‐sea) using the annotatr package based on the rn7 genome assembly. To avoid double counting in feature‐based summaries, a single primary genomic feature was assigned to each DMR using a predefined priority scheme (Promoter > Exon > Intron > Upstream 1–5 kb > 3′UTR > 5′UTR). Similarly, a primary CpG context was assigned per DMR using the order Island > Shore > Shelf > Open‐Sea. These primary annotations were used for mutually exclusive summary statistics and are provided in Table S1. Promoters were defined as the region 1 kb upstream to 500 bp downstream of the transcription start site. Genes associated with promoter, upstream regulatory, and gene‐body DMRs were analyzed separately to allow context‐specific interpretation. Functional enrichment analyses were performed using clusterProfiler for Gene Ontology Biological Process (GO‐BP) and KEGG pathway analysis. Enrichment significance was evaluated using Benjamini‐Hochberg‐adjusted *p* values, and terms meeting FDR ≤ 0.1 were considered significant.

### 2.10 Exploratory Correlation Analysis With Phenotype

As an exploratory analysis, Pearson correlations were performed between methylation levels of significant DMRs and individual VO_2_max values. Analyses were restricted to significant DMRs with valid gene annotation. VO_2_max correlation was performed using the RRBS quality‐passed samples (HCR *n* = 4, LCR *n* = 5). DMRs showing significant association with VO_2_max (FDR < 0.05) were retained for visualization.

### 2.11 Western Blotting and Protein Quantification

Cortical tissue was used for western blotting analyses. Selected epigenetic regulators, signaling proteins, synaptic proteins, inflammatory markers, and metabolic/mitochondrial proteins were assessed by Western blotting as previously described (Zhou, Mozaffaritabar, Koltai, et al. 2025). The analyzed targets included TET1 (PA5‐49432), TET2 (PA5‐85488), DNMT3A (PA5‐85548), DNMT3B (PA1‐884), phospho‐ERK1/2 (Thr202/Tyr204, 9106 s), total ERK1/2 (9102), phospho‐JNK (Thr183/185, 9251), total JNK2 (pa517634), p38 (9212), phospho‐mTOR (Ser2448, 5536), total mTOR (2983), phospho‐AKT (Ser473, 9271), total AKT (4691), phospho‐S6 (Ser235/236, 5364), total S6 (2217), phospho‐synapsin (Ser9, 2311), total synapsin (2312), synaptophysin (5461S), NF‐κB (3032), TNF‐α (sc1350), IκB‐α (Sc371), SOD2 (ab68155), OGG1 (15125‐1‐ap), PGC‐1α (NBP1‐04676), SIRT1 (ab110304), BDNF (ANT‐010), LDH (sc‐33781), OGDH (PA5‐28195), and SIRT3 (2627). Protein carbonylation was assessed using a Protein Carbonyl Assay Kit (ab178020). Total protein intensity (Ponceau S) was quantified using ImageJ for normalization. For phosphorylated proteins, membranes were first probed for the phosphorylated form, then stripped and re‐probed for total protein, with normalization performed on the same PVDF membrane. Western blot and protein carbonylation data were compared between LCR and HCR groups using unpaired Student's *t*‐tests (LCR, *n* = 6; HCR, *n* = 6). Vertical gaps separate non‐contiguous lanes originating from the same membrane.

## 3 Results

### 3.1 High Intrinsic Exercise Capacity Is Associated With Distinct DNA Methylation Patterns in the Aged Brain

**FIGURE 1:**
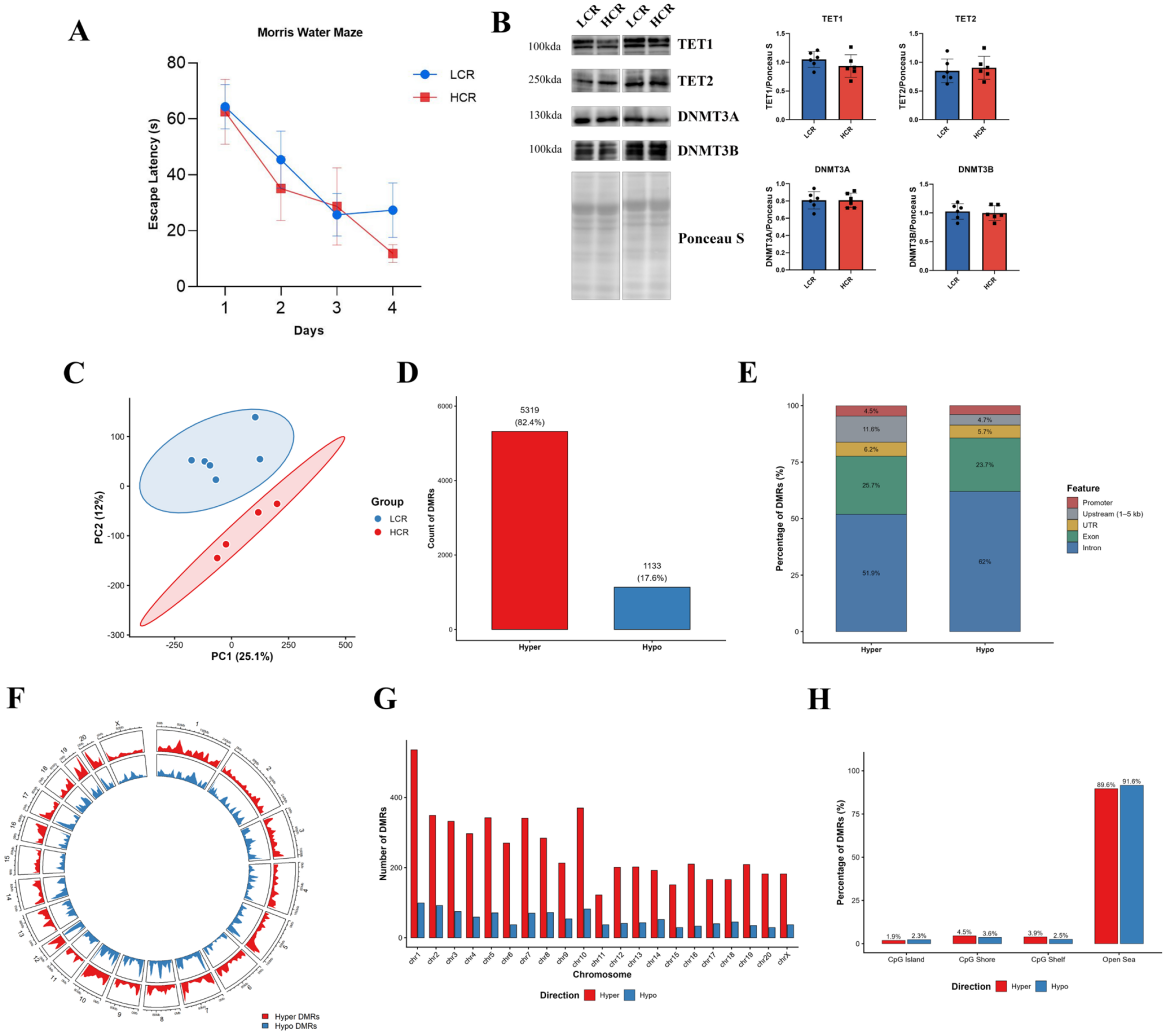
Behavioral, epigenetic‐regulatory, and DNA methylation features in aged HCR and LCR rat brains. (A) Morris water maze performance across 4 training days, shown as escape latency in LCR and HCR rats. Data are presented as mean ± SEM. (B) Representative Western blots and quantification of TET1, TET2, DNMT3A, and DNMT3B protein abundance in cortical samples. Data are presented as mean ± SD. (C) Principal component analysis (PCA) of hippocampal RRBS methylation data based on tested 1‐kb tiles. Each point represents one animal. (D) Numbers and percentages of hypermethylated and hypomethylated DMRs identified in HCR relative to LCR rats. (E) Distribution of gene‐associated DMRs across primary genomic features, including promoter, upstream (1–5 kb), UTR, exon, and intron. (F) Circos plot showing chromosomal distribution of DMRs, with hypermethylated DMRs in the outer track and hypomethylated DMRs in the inner track. (G) Chromosome‐wise counts of hypermethylated and hypomethylated DMRs. (H) Distribution of DMRs across CpG contexts, including CpG island, shore, shelf, and open‐sea.

As shown in Figure 1, we first assessed behavioral performance, methylation‐regulatory proteins, and RRBS‐based DNA methylation profiles in aged HCR and LCR rats. In the Morris water maze, both groups showed progressive reductions in escape latency across the 4‐day period, indicating learning in both groups (Figure 1A). No significant group difference was detected, although HCR rats exhibited slightly lower escape latencies. Given the limited number of aged animals in each group, this behavioral comparison may have been underpowered to detect moderate group differences. We next examined whether the methylation profile was accompanied by changes in major DNA methylation‐related enzymes. Cortical protein abundance of TET1, TET2, DNMT3A, and DNMT3B did not differ between groups (Figure 1B), suggesting that the observed methylation differences were not paralleled by detectable changes in these core methylation‐regulatory proteins.

PCA of RRBS methylation data showed clear separation between HCR and LCR samples, with PC1 and PC2 explaining 25.1% and 12.0% of the variance (Figure 1C). Differential methylation analysis identified 6452 significant DMRs, including 5319 hypermethylated DMRs (82.4%) and 1133 hypomethylated DMRs (17.6%) in HCR relative to LCR rats (Figure 1D, Table S1). Among gene‐associated DMRs, intronic and exonic regions represented the largest fractions, whereas promoter‐associated DMRs accounted for a smaller proportion (Figure 1E). DMRs were distributed across chromosomes, with hypermethylated regions outnumbering hypomethylated regions on each chromosome (Figure 1F,G). CpG context analysis showed that most DMRs were located in open‐sea regions (Figure 1H). Together, these findings indicate that high intrinsic exercise capacity is associated with a predominantly hypermethylated DMR profile enriched in open‐sea and gene‐body‐associated regions.

### 3.2 Hypermethylated DMR‐Associated Genes in HCR Brains Show Context‐Dependent Functional Enrichment

**FIGURE 2:**
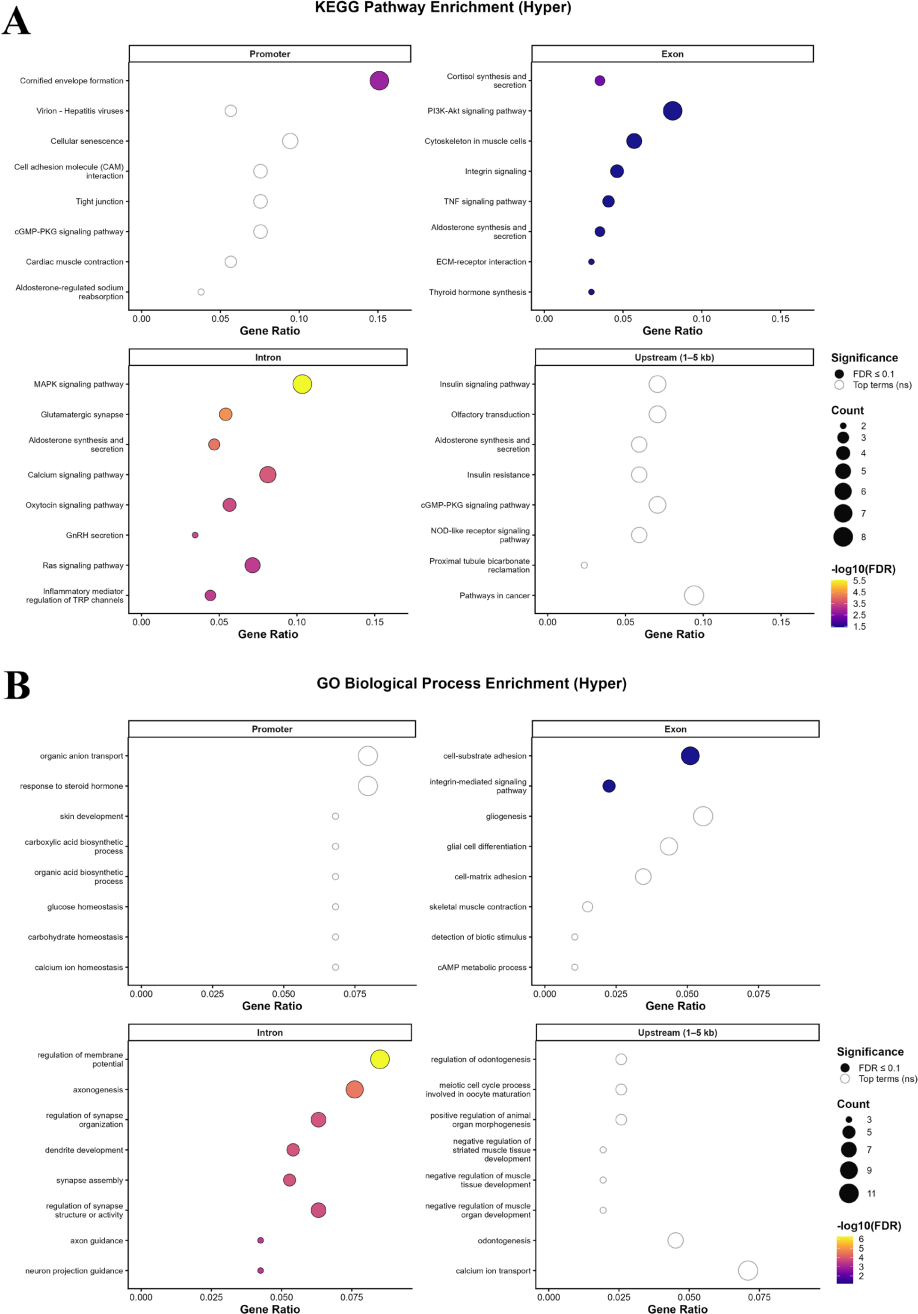
Functional enrichment analysis of hypermethylated DMR‐associated genes in aged HCR and LCR rat brains. (A) KEGG pathway enrichment analysis of genes associated with hypermethylated DMRs, stratified by primary genomic feature, including promoter, exon, intron, and upstream (1–5 kb) regions. (B) Gene Ontology Biological Process (GO‐BP) enrichment analysis of genes associated with hypermethylated DMRs, stratified by primary genomic feature, including promoter, exon, intron, and upstream (1–5 kb) regions. The *x*‐axis indicates gene ratio. Dot size represents gene count. Dot color indicates enrichment significance as −log_10_(FDR). Filled circles indicate terms meeting the significance threshold (FDR ≤ 0.1), whereas hollow circles represent top‐ranked terms that did not pass the FDR threshold but were retained for display.

To characterize the functional features of genes associated with hypermethylated DMRs, we performed KEGG and GO‐BP enrichment analyses stratified by primary genomic feature (Figure 2, Tables S2 and S3). This analysis revealed a feature‐dependent enrichment pattern, with the most evident signals observed among intron‐ and exon‐associated hypermethylated DMR genes.

In the KEGG analysis, intron‐associated hypermethylated DMR genes showed the most prominent enrichment profile, including MAPK signaling, glutamatergic synapse, calcium signaling, and Ras signaling, together with additional terms such as oxytocin signaling and GnRH‐related pathways. In parallel, GO Biological Process analysis of the same intronic gene set highlighted regulation of membrane potential, axonogenesis, dendrite development, regulation of synapse organization, synapse assembly, and axon guidance. Exon‐associated hypermethylated DMR genes also displayed significant enrichment. In KEGG analysis, the major enriched pathways included PI3K‐Akt signaling, TNF signaling, and ECM/integrin‐related pathways, whereas GO terms were mainly related to cell‐substrate adhesion and integrin‐mediated signaling pathways.

By contrast, promoter‐ and upstream‐associated hypermethylated DMR genes showed fewer FDR‐significant enriched terms. Several top‐ranked terms were retained for visualization, but only a small number reached the significance threshold. Overall, these findings suggest that the functional enrichment of hypermethylated DMR‐associated genes was more apparent in intronic and exonic regions. This context‐dependent enrichment pattern indicates that the main functional signals of hypermethylated DMR‐associated genes were concentrated in gene‐body and non‐promoter genomic contexts.

### 3.3 Protein Profiling Reveals Cortical Signaling and Inflammation‐Related Differences Aligned With DNA Methylation Enrichment in Aged HCR Rats

**FIGURE 3:**
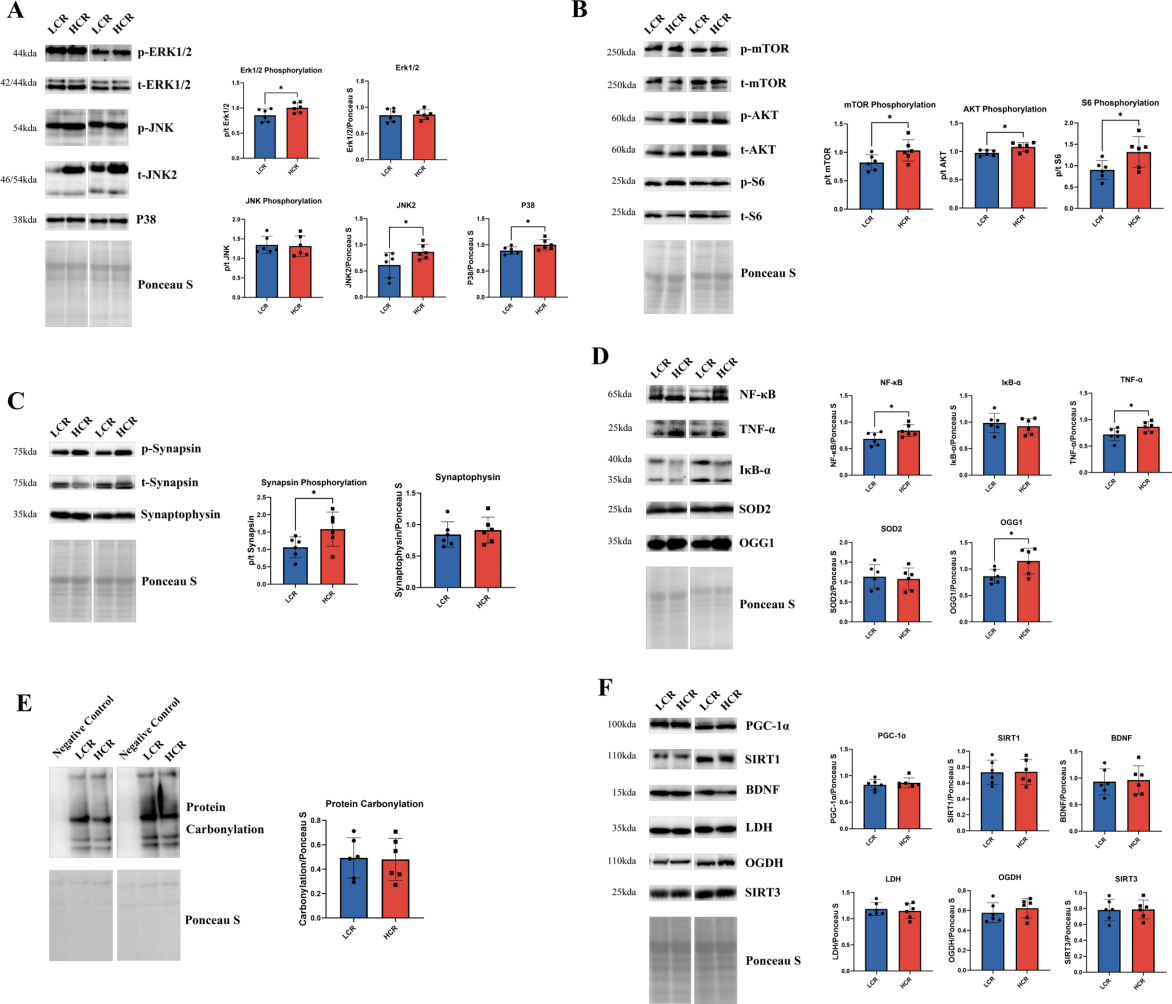
Cortical protein profiles in aged HCR and LCR rats. (A) Representative Western blots and quantification of phospho‐ERK1/2, total ERK1/2, phospho‐JNK, total JNK2, and p38. (B) Representative Western blots and quantification of phospho‐mTOR, total mTOR, phospho‐AKT, total AKT, phospho‐S6, and total S6. (C) Representative Western blots and quantification of phospho‐synapsin, total synapsin, and synaptophysin. (D) Representative Western blots and quantification of NF‐κB, TNF‐α, IκB‐α, SOD2, and OGG1. (E) Protein carbonylation blot and quantification. (F) Representative Western blots and quantification of PGC‐1α, SIRT1, BDNF, LDH, OGDH, and SIRT3. Data are presented as mean ± SD. *p < 0.05 between groups.

To assess whether the signaling pathways highlighted by DNA methylation enrichment were paralleled at the protein level, we examined selected cortical signaling, synaptic, inflammation‐related, oxidative stress, and metabolic markers by Western blotting (Figure 3). Within the MAPK‐related panel, ERK1/2 phosphorylation was higher in HCR rats, whereas total ERK1/2 abundance was unchanged (Figure 3A). JNK phosphorylation did not differ between groups, while total JNK2 and p38 abundances were higher in HCR rats, indicating selective differences in MAPK‐related signaling.

We next examined the AKT‐mTOR‐S6 axis, which was also highlighted by the enrichment analysis. HCR rats showed higher phosphorylation of mTOR, AKT, and S6 (Figure 3B), consistent with increased activity of this signaling axis. Among synaptic markers, synapsin phosphorylation was higher in HCR rats, whereas synaptophysin abundance was unchanged (Figure 3C), suggesting altered synaptic signaling rather than a broad increase in synaptic structural protein abundance. Because methylation enrichment also highlighted inflammation‐related pathways, we assessed inflammatory and stress‐responsive proteins. NF‐κB and TNF‐α abundance were higher in HCR rats, whereas IκB‐α and SOD2 did not differ between groups (Figure 3D). OGG1, a DNA repair‐related protein, was also higher in HCR rats. In contrast, protein carbonylation was unchanged (Figure 3E), suggesting that these inflammation‐ and DNA repair‐related changes were not accompanied by detectable increases in global protein oxidative damage. Finally, selected exercise‐responsive neurotrophic and metabolic/mitochondrial markers, including PGC‐1α, SIRT1, BDNF, LDH, OGDH, and SIRT3, did not differ between groups (Figure 3F).

Overall, these findings indicate that high intrinsic exercise capacity is associated with a cortical protein profile marked by enhanced MAPK‐, AKT‐mTOR‐S6‐, and synapsin‐related signaling, together with inflammation‐ and DNA repair‐related protein changes in the aged brain.

### 3.4 Exploratory Associations Between DMR Methylation and VO_2_max in Aged LCR and HCR Rats

**FIGURE 4:**
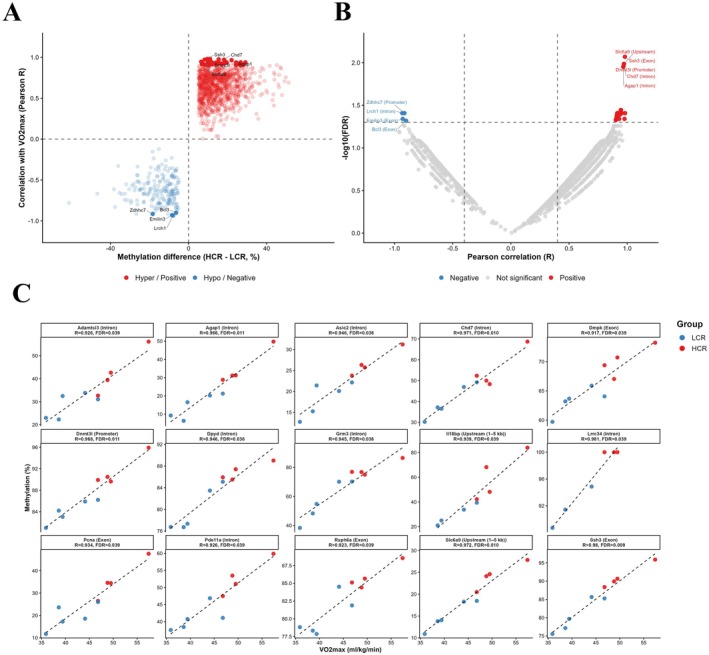
Exploratory associations between DMR methylation and VO_2_max in aged HCR and LCR rats. (A) Starburst plot showing the relationship between HCR–LCR methylation difference and Pearson correlation with VO_2_max for significant DMRs. (B) Correlation volcano plot showing Pearson correlation coefficients versus −log_10_(FDR) for DMR methylation–VO_2_max associations. Representative top positive and negative loci are labeled with gene symbol and primary genomic feature. (C) Scatter plots for representative top‐ranked VO_2_max‐associated DMRs, showing methylation level (%) versus VO_2_max for individual animals. Points are colored by group.

To explore whether hippocampal methylation variation was associated with aerobic performance in aged LCR and HCR rats, we correlated per‐animal methylation levels of significant DMRs with individual VO_2_max values. This exploratory analysis identified 46 VO_2_max‐associated DMRs at FDR < 0.05 (Figure 4, Table S4). These DMRs showed both positive and negative correlation directions and were mainly located in intronic and exonic regions (Figure 4A,B). Representative positively correlated loci were annotated to Ssh3, Slc6a9, Chd7, Dnmt3l, and Agap1, whereas negatively correlated loci included Lrch1, Zdhhc7, Emilin3, and Bcl3 (Figure 4B,C). Given the limited sample size, these exploratory results should be interpreted as VO_2_max‐aligned candidate methylation regions that require validation in future studies.

## 4 Discussion

The present study suggests that high intrinsic exercise capacity in aged rats is associated with a distinct brain molecular phenotype involving DNA methylation differences and a parallel protein signaling profile. Compared with LCR animals, the aged HCR hippocampus showed a distinct and context‐dependent DMR profile, characterized by a higher proportion of hypermethylated DMRs and enrichment in open‐sea CpG and gene‐body contexts. Functional enrichment of hypermethylated DMR‐associated genes highlighted pathways related to MAPK, calcium, PI3K‐Akt, TNF, and synaptic regulation. In parallel, cortical protein profiling showed higher phosphorylation of ERK1/2, AKT‐mTOR‐S6, and synapsin‐related signaling proteins, together with higher abundance of inflammation‐ and DNA repair‐related proteins. Together, these findings indicate that high intrinsic aerobic capacity is linked to a biologically complex aged brain molecular profile rather than a uniformly protective phenotype.

One notable feature of the present methylation dataset is that the HCR‐associated DMR profile was predominantly hypermethylated and preferentially located in open‐sea CpG regions and gene‐body features, particularly intronic and exonic regions. Previous exercise intervention studies have shown that physical activity can modulate brain epigenetic regulation, often through promoter‐level changes in plasticity‐related genes such as Bdnf, together with changes in DNMT/TET‐related pathways (Zheng et al. 2025). By contrast, the pattern observed in the present study suggests that aerobic capacity is associated less with a promoter‐centered model and more with a gene‐body‐enriched methylation pattern (Bittel and Chen 2024; Neri et al. 2017). Such patterns are consistent with accumulating evidence that brain DNA methylation is not confined to promoter regions, but also extends to gene bodies and distal regulatory elements, where it may contribute to long‐term regulatory programs (Bittel and Chen 2024; Etayo‐Urtasun et al. 2024; Halder et al. 2016; Maasar et al. 2021; Neri et al. 2017; Vasileva et al. 2024; Zhou, Mozaffaritabar, Tanisawa, et al. 2025). One important question is how the gene‐body hypermethylation should be interpreted. Unlike promoter methylation, which is generally associated with transcriptional repression, gene‐body methylation is often enriched within actively transcribed genes and may contribute to transcriptional fidelity by limiting spurious intragenic transcription initiation (Neri et al. 2017). Therefore, the hypermethylated DMRs observed in HCR rats should not be interpreted simply as markers of gene silencing. An additional observation is that the core DNA methylation‐related enzymes TET1/2 and DNMT3A/B were unchanged. Thus, the HCR‐associated methylation profile was not paralleled by detectable changes in these canonical methylation‐ or demethylation‐related enzymes (Zhou, Mozaffaritabar, Tanisawa, et al. 2025).

The context‐dependent enrichment analysis further supports the importance of non‐promoter hypermethylated DMRs in the HCR‐associated methylation profile. In particular, intronic and exonic hypermethylated DMR‐associated genes were enriched for pathways related to MAPK signaling, calcium signaling, synaptic regulation, PI3K‐Akt signaling, TNF signaling, and cell‐adhesion processes. These pathway‐level findings were broadly aligned with the cortical protein profile, particularly the MAPK‐related changes, including higher ERK1/2 phosphorylation and increased p38 and JNK2 abundance, together with higher AKT‐mTOR‐S6 signaling, synapsin phosphorylation, and inflammation‐related protein abundance. Together, these findings suggest a pathway‐level alignment between the HCR‐associated methylation profile and cortical protein signaling changes.

An important conceptual implication of this study is that the aged HCR brain phenotype should not be interpreted simply as “better” or uniformly protected. On the one hand, higher phosphorylation of ERK1/2, AKT‐mTOR‐S6 axis components, and synapsin points to greater engagement of pathways related to synaptic integrity and plasticity, protein synthesis, and structural remodeling (Cesca et al. 2010; Halder et al. 2016; Lister et al. 2013; Radak et al. 2008; Thomas and Huganir 2004). On the other hand, HCR rats also showed higher abundance of JNK2, p38, NF‐κB, TNF‐α, and OGG1, indicating concomitant activation of stress‐, inflammation‐, and DNA repair‐related processes (Glass et al. 2010; Radak et al. 2024). This is relevant to our previous findings showing that lifelong skeletal muscle PGC‐1α overexpression increased running capacity in aged mice while simultaneously compromising brain health and promoting oxidative stress and inflammation (Zhou, Mozaffaritabar, Koltai, et al. 2025). In the present study, inherited high aerobic capacity was likewise associated with an inflammatory signature in the aged brain. It is also crucial to highlight that some studies have shown that physiological elevations of these stress kinases and cytokines (TNF, NF‐κB, JNK, p38) are also obligatory for synaptic scaling and hormetic adaptation (Coffey 2014; Santello and Volterra 2012), acting as double‐edged swords. Importantly, several canonical markers commonly associated with exercise‐induced neuroadaptation, including PGC‐1α, SIRT1, BDNF, SOD2, and SIRT3, were unchanged between groups (Wrann et al. 2013; Zhou et al. 2022). This dissociation highlights a fundamental distinction between intrinsic aerobic capacity and exercise intervention, suggesting that inherited fitness does not recapitulate the classical adaptation induced by training.

This mixed phenotype is important when interpreting the behavioral results. Although HCR rats tended to show lower escape latencies in the Morris water maze, the group difference was not statistically significant. Thus, the molecular differences observed between HCR and LCR brains did not translate into a clearly superior spatial learning phenotype in this assay. This may reflect the limited behavioral sample size, the high variability commonly observed in aged animals, or the possibility that increased signaling activity does not necessarily produce measurable cognitive improvement when inflammation‐ and stress‐related pathways are also elevated.

Exploratory VO_2_max correlation analysis provided an additional association layer between hippocampal methylation variation and aerobic performance in aged LCR and HCR rats. These loci included both positive and negative associations and were mainly annotated to gene‐body regions. Representative loci were linked to genes with potential relevance to synaptic signaling, neuronal plasticity, epigenetic regulation, membrane trafficking, and inflammation‐related transcriptional regulation (Glass et al. 2010; Halder et al. 2016; Neri et al. 2017). Given the limited sample size, these DMRs should be interpreted as candidate aerobic capacity‐associated methylation regions requiring future validation.

Several limitations should be acknowledged. First, the RRBS sample size was reduced after quality control, leaving a relatively small number of animals for methylation analyses. Second, the study was cross‐sectional and therefore does not establish causality. Third, RRBS and protein measurements were performed in different brain regions, with methylation assessed in the hippocampus and protein signaling examined in the cortex; therefore, the methylation–protein relationship should be interpreted as pathway‐level evidence rather than as direct region‐matched validation. Fourth, matched transcriptomic data and locus‐specific functional validation were not available, limiting direct interpretation of the regulatory consequences of the identified DMRs. Finally, only aged female rats were studied, and potential sex‐specific effects remain unknown.

## 5 Conclusion

In conclusion, high intrinsic exercise capacity in aged rats is associated with a distinct brain molecular profile characterized by hippocampal hypermethylation‐enriched DMR patterns, preferential localization to open‐sea and gene‐body regions, and parallel cortical signaling changes involving MAPK‐, AKT‐mTOR‐S6‐, synaptic‐, inflammation‐, and DNA repair‐related pathways. Rather than supporting a uniformly protective model, these findings suggest that intrinsic aerobic capacity is linked to a biologically complex aged brain state in which neuroplasticity‐related signaling and stress‐responsive features coexist. These results provide candidate methylation regions and signaling pathways for future studies investigating how fitness‐related traits influence brain aging.

## Author Contributions

L.Z. performed the data analysis, generated the figures, and drafted the manuscript; L.Z. and S.M. carried out the Western blot experiments; L.Z., E.K., and T.K. conducted the animal experiments, performed the VO_2_max testing; M.H., Y.G., and S.K. assisted with data interpretation; L.G.K. and S.L.B. provided the HCR/LCR rat models; Z.R. supervised the study and critically revised the manuscript. All authors have read and approved the final version of the manuscript.

## Funding

ZR acknowledges support from the National Science and Research Fund (OTKA142192) and HU‐RIZONT‐2025‐00096.

## Conflicts of Interest

The authors declare no conflicts of interest.

## Supporting information


**Table S1:** Significant differentially methylated regions between aged HCR and LCR rat hippocampal tissue. This table lists all significant differentially methylated regions (DMRs) identified by RRBS analysis between high‐capacity runner (HCR) and low‐capacity runner (LCR) rats. DMRs were defined using 1‐kb tiling windows with FDR < 0.10 and methylation difference > 5%. The table includes genomic coordinates, methylation difference, *p* value, *q* value, methylation direction, mean methylation levels in HCR and LCR groups, primary genomic feature, CpG context, and associated gene annotation. Positive methylation differences indicate hypermethylation in HCR relative to LCR, whereas negative values indicate hypomethylation in HCR relative to LCR.

**Table S2:** Gene Ontology Biological Process enrichment of DMR‐associated genes. This table provides Gene Ontology Biological Process enrichment results for genes associated with significant DMRs, stratified by methylation direction and primary genomic feature. Enrichment terms meeting FDR ≤ 0.10 are shown, including GO term ID, description, gene ratio, background ratio, enrichment statistics, adjusted *p* value, *q* value, associated genes, gene count, methylation direction, and genomic feature category.

**Table S3:** KEGG pathway enrichment of DMR‐associated genes. This table provides KEGG pathway enrichment results for genes associated with significant DMRs, stratified by methylation direction and primary genomic feature. Enriched pathways meeting FDR ≤ 0.10 are shown, including pathway category, subcategory, KEGG ID, pathway description, gene ratio, background ratio, enrichment statistics, adjusted *p* value, *q* value, associated genes, gene count, methylation direction, and genomic feature category.

**Table S4:** Significant correlations between DMR methylation levels and VO_2_max. This table lists DMRs whose methylation levels showed significant Pearson correlations with individual VO_2_max values (FDR ＜ 0.10). The table includes correlation coefficient, *p* value, FDR, DMR ID, methylation direction, primary genomic feature, associated gene symbols, methylation difference, q value, and feature annotation. Positive correlations indicate DMRs whose methylation levels were positively associated with VO_2_max, whereas negative correlations indicate inverse associations.

## Data Availability

Raw and processed methylation data are publicly available in the Gene Expression Omnibus (GEO) under accession number GSE328395.
